# Supplementary material for: Attitudes to the use of animals in biomedical research: Effects of stigma and selected research project summaries
Source: PLoS One. 2023 Aug 18;18(8):e0290232. doi: 10.1371/journal.pone.0290232 (PMC10437917; doi:10.1371/journal.pone.0290232)
Supplement: S1 Appendix — (DOCX) [file pone.0290232.s001.docx]

**Pause for thought: The role of corticostriatal circuitry and its dopamine innervation in the inhibitory modulation of associative learning**

**https://gtr.ukri.org/projects?ref=BB%2FS000119%2F1**

**Lay summary length matched to technical**

The nuts and bolts of everyday thinking, provided by the formation of connections between related events, enable us to establish clear trains of thought. Associative learning mechanisms, common to all animals, support not only the cueing of thoughts, words and deeds, but also the ability to hesitate through the use of a 'mental brake' to suppress inappropriate thoughts and actions. When the ability to restrain the impulse generated by an association is lost, diverse symptoms may result. Clinically, impaired inhibitory modulation has been identified as contributing to a number of disorders, including addiction, anxiety, obesity and schizophrenia. Learning procedures developed in the laboratory rat provide an excellent model system to study the gating of unwanted associations. If a particular event predicts an outcome, learning is normally demonstrated by the animal's behavioural reaction to the first event. However, if the first event is presented in conjunction with another cue which means that the expected outcome will not now occur, the normal behavioural and cognitive reactions are inhibited. Impairments in such inhibition could explain a variety of symptoms, from over-eating when the consequences of eating more food will no longer be pleasant, to some of the disordered thought patterns identified with schizophrenia. The present project will advance previous findings in that we will selectively interfere with chemical signalling in specific brain pathways, by targeted drug delivery to areas first identified by temporarily inactivating small brain regions. Importantly, the behavioural procedures to be used in this project work in humans too; although the experimental details differ, there is sufficient similarity to translate findings from the animal laboratory to the clinic (and vice versa). Experiments in rats delineate the brain substrates of inhibitory learning and give vital clues as to where and how new treatments should work, with minimum side-effects.
[296 words]

**Technical summary**

Conditioned inhibition is a form of learning seen when an otherwise expected event does not occur in the presence of the inhibitor. Such inhibitory modulation is fundamental to many aspects of normal psychological function, such as the control of food intake, while impairments in this process underpin a wide variety of mental health conditions. We have found that this kind of inhibitory learning is impaired in humans with schizophrenia (as well as those with particular personality profiles) and have adapted the experimental design for use in rats, with a refined appetitive procedure, to investigate the role of the dopamine (DA) system and interconnected cortical structures, specifically medial prefrontal cortex (mPFC). We will use this translational task to analyse the role of corticostriatal DA in inhibitory modulation, with a view to developing novel therapeutic strategies. The mPFC and DA systems have been independently identified as being involved in aspects of inhibition. The proposed plan of work will advance on these findings by testing for dissociable effects in mPFC sub-regions. Moreover, the mPFC projects topographically to nucleus accumbens (NAc) in the striatum, which is also a functionally heterogenous structure. We will compare the effects of (1) regional inactivation and (2) electrophysiological profiles in NAc and the corresponding sub-regions in mPFC, to identify functional interactions between the two structures. We will go on to investigate the role of particular DA receptor sub-types in a series of micro-infusion studies at the coordinates identified in the regional inactivation studies. This will allow us to examine the modulatory role of DA in the key brain regions of interest. The proposed use of a translational task in a rat model allows for localised interventions (including disconnection studies using crossed unilateral infusions) to establish cause and effect, combined with correlational studies of interconnectivity of small sub-regions.

[298 words]
