# Supplementary material for: Attitudes to the use of animals in biomedical research: Effects of stigma and selected research project summaries
Source: PLoS One. 2023 Aug 18;18(8):e0290232. doi: 10.1371/journal.pone.0290232 (PMC10437917; doi:10.1371/journal.pone.0290232)
Supplement: S3 Appendix — (DOCX) [file pone.0290232.s003.docx]

1. **Pairwise comparisons for species by summary**

Survey Species 1 Species 2 Difference (std. error) p-value^b^

no summary pig rat .555* (.051) <.001

mouse .467* (.050) <.001

fish .303* (.056) <.001

monkey -.257* (.047) <.001

rat pig -.555* (.051) <.001

mouse -.087* (.024) .003

fish -.251* (.051) <.001

monkey -.812* (.064) <.001

mouse pig -.467* (.050) <.001

rat .087* (.024) .003

fish -.164* (.050) .011

monkey -.724* (.062) <.001

fish pig -.303* (.056) <.001

rat .251* (.051) <.001

mouse .164* (.050) .011

monkey -.560* (.070) <.001

monkey pig .257* (.047) <.001

rat .812* (.064) <.001

mouse .724* (.062) <.001

fish .560* (.070) <.001

lay summary pig rat .364* (.055) <.001

mouse .328* (.054) <.001

fish .282* (.061) <.001

monkey -.225* (.051) <.001

rat pig -.364* (.055) <.001

mouse -.036 (.026) 1.000

fish -.082 (.056) 1.000

monkey -.589* (.070) <.001

mouse pig -.328* (.054) <.001

rat .036 (.026) 1.000

fish -.047 (.054) 1.000

monkey -.554*(.067) <.001

fish pig -.282* (.061) <.001

rat .082 (.056) 1.000

mouse .047 (.054) 1.000

monkey -.507* (.076) <.001

monkey pig .225* (.051) <.001

rat .589* (.070) <.001

mouse .554* (.067) <.001

fish .507* (.076) <.001

technical summary pig rat .315* (.052) <.001

mouse .259* (.052) <.001

fish .148 (.058) .115

monkey -.163* (.049) .009

rat pig -.315* (.052) <.001

mouse -.056 (.025) .244

fish -.167* (.053) .018

monkey -.478* (.066) <.001

mouse pig -.259* (.052) <.001

rat .056 (.025) .244

fish -.111 (.052) .330

monkey -.422* (.064) <.001

fish pig -.148 (.058) .115

rat .167* (.053) .018

mouse .111 (.052) .330

monkey -.311* (.072) <.001

monkey pig .163* (.049) .009

rat .478* (.066) <.001

mouse .422* (.064) <.001

fish .311* (.072) <.001

Note: Based on estimated marginal means.* The mean difference is significant at the .05 level; non-significant comparisons are highlighted. ^b^Adjustment for multiple comparisons: Bonferroni.

1. **Pairwise comparisons for species by disorder**

Disorder Species 1 Species 2 Difference (std. error) p-value^b^

addiction pig rat .519* (.036) <.001

mouse .466* (.036) <.001

fish .310* (.037) <.001

monkey -.153* (.033) <.001

rat pig -.519* (.036) <.001

mouse -.053 (.019) .051

fish -.209* (.037) <.001

monkey -.672* (.043) <.001

mouse pig -.466* (.036) <.001

rat .053 (.019) .051

fish -.156* (.036) <.001

monkey -.619* (.042) <.001

fish pig -.310* (.037) <.001

rat .209* (.037) <.001

mouse .156* (.036) <.001

monkey -.463* (.046) <.001

monkey pig .153* (.033) <.001

rat .672* (.043) <.001

mouse .619* (.042) <.001

fish .463* (.046) <.001

schizophrenia pig rat .460* (.035) <.001

mouse .411* (.035) <.001

fish .295* (.038) <.001

monkey -.130* (.033) .001

rat pig -.460* (.035) <.001

mouse -.049 (.019) .092

fish -.165* (.035) <.001

monkey -.590* (.043) <.001

mouse pig -.411* (.035) <.001

rat .049 (.019) .092

fish -.116* (.035) .011

monkey -.541* (.043) <.001

fish pig -.295* (.038) <.001

rat .165* (.035) <.001

mouse .116* (.035) .011

monkey -.425* (.047) <.001

monkey pig .130* (.033) .001

rat .590* (.043) <.001

mouse .541* (.043) <.001

fish .425* (.047) <.001

obesity pig rat .346* (.036) <.001

mouse .251* (.035) <.001

fish .195* (.038) <.001

monkey -.298* (.035) <.001

rat pig -.346* (.036) <.001

mouse -.095* (.022) <.001

fish -.152* (.034) <.001

monkey -.644* (.042) <.001

mouse pig -.251* (.035) <.001

rat .095* (.022) <.001

fish -.056 (.035) 1.000

monkey -.549* (.041) <.001

fish pig -.195* (.038) <.001

rat .152* (.034) <.001

mouse .056 (.035) 1.000

monkey -.492* (.045) <.001

monkey pig .298* (.035) <.001

rat .644* (.042) <.001

mouse .549* (.041) <.001

fish .492* (.045) <.001

CVD pig rat .319* (.035) <.001

mouse .278* (.036) <.001

fish .178* (.038) <.001

monkey -.279* (.035) <.001

rat pig -.319* (.035) <.001

mouse -.041 (.019) .279

fish -.141* (.034) <.001

monkey -.599* (.044) <.001

mouse pig -.278* (.036) <.001

rat .041 (.019) .279

fish -.100* (.033) .030

monkey -.558* (.043) <.001

fish pig -.178* (.038) <.001

rat .141* (.034) <.001

mouse .100* (.033) .030

monkey -.458* (.045) <.001

monkey pig .279* (.035) <.001

rat .599* (.044) <.001

mouse .558* (.043) <.001

fish .458* (.045) <.001

Note: Based on estimated marginal means.* The mean difference is significant at the .05 level; non-significant comparisons are highlighted. ^b^Adjustment for multiple comparisons: Bonferroni. CVD = cardiovascular disease.

1. **Pairwise comparisons for species by gender**

Gender Species 1 Species 2 Difference (std. error) p-value^b^

male pig rat .284* (.057) <.001

mouse .194* (.057) .007

fish .188* (.064) .034

monkey -.269* (.053) <.001

rat pig -.284* (.057) <.001

mouse -.091* (.027) .010

fish -.097 (.058) .947

monkey -.553* (.072) <.001

mouse pig -.194* (.057) .007

rat .091* (.027) .010

fish -.006 (.056) 1.000

monkey -.463* (.069) <.001

fish pig -.188* (.064) .034

rat .097 (.058) .947

mouse .006 (.056) 1.000

monkey -.457* (.079) <.001

monkey pig .269* (.053) <.001

rat .553* (.072) <.001

mouse .463* (.069) <.001

fish .457* (.079) <.001

female pig rat .463* (.039) <.001

mouse .406* (.038) <.001

fish .283* (.043) <.001

monkey -.184* (.036) <.001

rat pig -.463* (.039) <.001

mouse -.057* (.018) .019

fish -.181* (.039) <.001

monkey -.647* (.048) <.001

mouse pig -.406* (.038) <.001

rat .057* (.018) .019

fish -.123* (.038) .012

monkey -.590* (.046) <.001

fish pig -.283* (.043) <.001

rat .181* (.039) <.001

mouse .123* (.038) .012

monkey -.467* (.053) <.001

monkey pig .184* (.036) <.001

rat .647* (.048) <.001

mouse .590* (.046) <.001

fish .467* (.053) <.001

Note: Based on estimated marginal means.* The mean difference is significant at the .05 level; non-significant comparisons are highlighted. ^b^Adjustment for multiple comparisons: Bonferroni.

1. **Pairwise comparisons for species by degree level education and psychological (vs physical) disorder**

*Relevant degree*

Disorder Species 1 Species 2 Difference (std. error) p-value^b^

psychological pig rat .656* (.058) <.001

mouse .610* (.058) <.001

fish .403* (.065) <.001

monkey -.141 (.054) .091

rat pig -.656* (.058) <.001

mouse -.047 (.030) 1.000

fish -.254* (.060) <.001

monkey -.798* (.070) <.001

mouse pig -.610* (.058) <.001

rat .047 (.030) 1.000

fish -.207* (.059) .005

monkey -.751* (.069) <.001

fish pig -.403* (.065) <.001

rat .254* (.060) <.001

mouse .207* (.059) .005

monkey -.544* (.079) <.001

monkey pig .141 (.054) .091

rat .798* (.070) <.001

mouse .751* (.069) <.001

fish .544* (.079) <.001

physical pig rat .404* (.058) <.001

mouse .352* (.058) <.001

fish .252* (.063) <.001

monkey -.313* (.055) <.001

rat pig -.404* (.058) <.001

mouse -.052 (.030) .856

fish -.152 (.055) .059

monkey -.717* (.071) <.001

mouse pig -.352* (.058) <.001

rat .052 (.030) .856

fish -.100 (.054) .659

monkey -.665* (.068) <.001

fish pig -.252* (.063) <.001

rat .152 (.055) .059

mouse .100 (.054) .659

monkey -.565* (.075) <.001

monkey pig .313* (.055) <.001

rat .717* (.071) <.001

mouse .665* (.068) <.001

fish .565* (.075) <.001

*No relevant degree*

psychological pig rat .391* (.042) <.001

mouse .329* (.042) <.001

fish .259* (.046) <.001

monkey -.128* (.039) .010

rat pig -.391* (.042) <.001

mouse -.062* (.021) .041

fish -.132* (.043) .021

monkey -.519* (.050) <.001

mouse pig -.329* (.042) <.001

rat .062* (.021) .041

fish -.070 (.042) .955

monkey -.457* (.049) <.001

fish pig -.259* (.046) <.001

rat .132* (.043) .021

mouse .070 (.042) .955

monkey -.387* (.057) <.001

monkey pig .128* (.039) .010

rat .519* (.050) <.001

mouse .457* (.049) <.001

fish .387* (.057) <.001

physical pig rat .279* (.042) <.001

mouse .195* (.041) <.001

fish .154* (.045) .006

monkey -.252* (.039) <.001

rat pig -.279* (.042) <.001

mouse -.084* (.022) .001

fish -.125* (.040) .017

monkey -.531* (.051) <.001

mouse pig -.195* (.041) <.001

rat .084* (.022) .001

fish -.040 (.039) 1.000

monkey -.447* (.049) <.001

fish pig -.154* (.045) .006

rat .125* (.040) .017

mouse .040 (.039) 1.000

monkey -.407* (.054) <.001

monkey pig .252* (.039) <.001

rat .531* (.051) <.001

mouse .447* (.049) <.001

fish .407* (.054) <.001

Note: Based on estimated marginal means.* The mean difference is significant at the .05 level; non-significant comparisons are highlighted. ^b^Adjustment for multiple comparisons: Bonferroni.
