# Supplementary material for: Attitudes to the use of animals in biomedical research: Effects of stigma and selected research project summaries
Source: PLoS One. 2023 Aug 18;18(8):e0290232. doi: 10.1371/journal.pone.0290232 (PMC10437917; doi:10.1371/journal.pone.0290232)
Supplement: Attachment — Submitted filename: Attitudes to the use of animals in biomedical research.docx [file pone.0290232.s004.docx]

Attitudes to the use of animals in biomedical research: Effects of stigma and selected research project summaries

This manuscript examines attitudes towards the use of animals in biomedical research, specifically the effects of the reading research summaries (none, lay, technical) on expressed attitudes towards animals in biomedical research. The effect of the controllability of the disorder being researched was varied, and consistency across different animal species was also examined. The topic is a very interesting one, and the study has a lot of potential. As it currently stands it needs quite a bit of editing, particularly in the Introduction, Methods, and Discussion sections to more clearly convey the rationale for the study and why the work is important. In addition, the hypotheses as currently stated are simple, but the design is a 4-way mixed design; the hypotheses should reflect this complexity. There are also methodological aspects of the study that need to be clarified, including whether participants were randomised to the between-subjects conditions and whether any of the materials were piloted beforehand.

Abstract

- Needs to be edited for clarity – some info about methods shared mid-way through the abstract, making it harder to differentiate what is methods/results. The abstract doesn’t make it clear how stigma is related to the research that was conducted
- Were the summaries presented between-groups? What about disorder types and animal species? Edit to make this clearer
- The final sentence about findings could be more impactful – instead of saying “found effects of summary” comment on whether type of information about a study affects attitudes to use of animals in biomedical research.

Introduction

- Pg 3 – is it barriers to general public/lay understanding being examined? Suggest state explicitly
- Pg 4 – paragraph on obesity stands out on its own, unclear how it links to the overarching discussion of stigma and mental illness; this section needs an overarching sentence(s) to tie together the relevance of stigma to the study aims – this link wasn’t clear to me from what is written. Having read more of the paper I now know that you will be using obesity as one of the conditions – the reader doesn’t know this yet in the intro, so the relevance needs to be explicitly stated/worked into the thread of the argument
- Animal species section needs to be tied back in to overarching study aims
- Not clear to me from what is written how stigma is related to the effect of different ways of describing studies to lay people and how that relates to attitudes towards biomedical research. This needs to be explicit
- There isn’t much in the introduction about what is currently known about attitudes. While there may not be much research specifically looking at how information is presented (e.g., in summaries) and how that impacts attitudes, there is work looking at attitudes to biomedical research more generally that could be discussed
- Section “Measuring effects of selected research project summaries” (p 5) reads like a methods section. I’m not certain this section contributes to the overall argument of the Introduction
- Aims and Hypotheses- too much of this section reads as a methods section. This needs to be more about the constructs, rather than describing what was done.
- The 3 predictions should be more specific, and include comparison groups. Phrases like “should reduce”, “should be relatively more support” need to be tightened up. Having read further in the paper, the hypotheses outlined in the Introduction are very simple compared to the very complex 3x5x2x2 design outlined on p14. The hypotheses need re-writing to match the complexity of the study design and expected findings

Methods

- How were participants allocated to the summary groups? Was it random? When you say “different versions of the survey were not available at the same time” does this refer to the different summary groups?
- Unclear why ANOVAs are referred to in the participants section
- Description in Materials needs to be more specific. For example, What is ‘eating preference’ referred to on p9? Is it the same as ‘eating orientation’ on p11. Suggest that you are specific about the number of questions rather than saying phrases such as “a few additional questions”
- Measures – what is the range of possible scores for each measure
- There is some repetition of information within the method
- Information such as diseases designated controllable vs uncontrollable, and the physical vs psychological should be introduced prior to the ‘Data analysis’ section as it’s the design of the study
- It is unclear whether the summaries were piloted – were they in fact lay vs technical in the view of the general public?

Results

- This study is trying to do a lot all in one, and as a result it’s difficult to determine what the key results are. The complexity of the study design means clarity of writing and presentation of information is extra important.
- Headings need to be clear and be linked to the constructs being examined, not just the survey components. For example the heading “Overall differences between the summary variants of the surveys” seems to be describing differences in participant characteristics across the summary type conditions
- The clarity of results displayed in charts would be increased having numbers on top of the bars on the charts to show the means. The mean differences are not very large, so it would be helpful to see the specific numbers when interpreting results.
- Table 3 title is ‘Differences in ratings’ are these mean differences? It would be good to see the means themselves, not just differences.
- When interactions were found to be non-significant were they removed from the model and the analyses re-run to examine the relationships of interest?

Discussion

This section needs harmonising with the introduction. Concepts and relationships discussed here aren’t always clearly introduced in the Introduction, meaning that the discussion of results isn’t as strong as it could be.

- Some parts read more as a summary of results than an interpretation with links to existing research (for example, p23, paragraph 3)
- The discussion of CVD as potentially being perceived as controllable (p25) seems to me to be something that should be included in the limitations section. Was controllability piloted? I don’t remember reading that it was.
- There was not as much discussion of the lay summary vs no summary vs technical summary differences as I would have expected. I would have thought that the no summary group gives you an idea of people’s general attitudes and a useful comparison for examining any effects of summary.

Other

- Figure 1 appears to contain both an example of a survey question and study results?
- Figures 1 & 2 – lower bound of the CI?SE? isn’t visible on some of the colours (also suggest a note clarifying what the bars on the charts represent)
