# Supplementary material for: Attitudes to the use of animals in biomedical research: Effects of stigma and selected research project summaries
Source: PLoS One. 2023 Aug 18;18(8):e0290232. doi: 10.1371/journal.pone.0290232 (PMC10437917; doi:10.1371/journal.pone.0290232)
Supplement: Attachment — Submitted filename: Response to Reviewers.docx [file pone.0290232.s006.docx]

**Responses to reviewers**

*We thank the Editor and Reviewer for their constructive comments to further improve the manuscript. The points raised are addressed below and revisions to the manuscript are highlighted. Our own minor corrections are also highlighted.*

**Editor**

*Response:* *We have addressed Reviewer #1’s further comments as required (please see below). Reviewer #2 confirms that all comments have been addressed. I confirm that we submitted the data as a supplementary file which we assumed should have been available to reviewers. I put an embargo on the University of Nottingham repository link as we prefer to publish the findings before we share the underpinning data without any restriction. There is still a difference of opinion on the use of multiple imputation for missing data, but Reviewer 2 is overall satisfied.*

**Reviewer #1**

Introduction
• Some parts of the Introduction still need to be tightened up to more clearly tie the material presented to the study aims and hypotheses. For example, in the section discussing Stigma and blame (p5) there is a paragraph with general information about stigma, then stereotypes about people with mental illness, then biases towards specific disorders, followed by a short paragraph describing obesity stigma. As a reader I was left wondering why this information was being presented, and how it connected to the animal species section presented below it. I suggest combining paragraphs and adding in sentences tying the information presented to the aims and hypotheses to strengthen the argument being presented in the introduction.

*Response: The Introduction has been tightened up as suggested.*

Methods
• P10 suggest the change the phrasing of “(to avoid the possibility of responses to more than one survey)” to “reduce the possibility…” as it remains possible that people use more than one social network, so the risk can’t be avoided altogether.

*Response: This has been done.*

• P10 it’s not clear to me what a matched social network would be

*Response: The social networks were judged to be similar based on the similar demographics of the researchers, but we agree that we cannot really claim that they were ‘matched’ in any formal sense. The wording has been revised accordingly.*

• On p11 in the design section the predictions are referred to as being both “key” and “general” – I don’t recall any key predictions being made in the introduction. Suggest using a consistent term.

*Response: We have removed this unhelpful distinction and now consistently refer to predictions.*

Results
• P23, “largest p = 0.019 for the difference between” if the authors are aiming to comment on the largest effect, then it would be preferrable to compare effect sizes rather than p-values (this also applies elsewhere in the Results).

*Response: The Results presentation has been edited to avoid this issue. We now simply refer to the S3 Appendix for full details of the pairwise comparisons. We have also added a couple of missing effect sizes for the ANOVAs.*

Discussion
• The first line of the discussion (p25) “The APQ ratings, which were…” would be strengthened by referring to the construct itself, rather than to APQ ratings (which tap into the construct of interest)

*Response: The opening to the Discussion has been re-phrased.*

• P25 “and there was no dramatic difference in the profiles of responses” unclear to me what kind of difference would be considered “dramatic”; suggest re-wording

*Response: This statement has been re-worded.*

• In the manuscript “e.g.” is often used within a sentence (e.g., p32 “…wider range of disorders, e.g. depression and anxiety…”), the convention is typically e.g., within parentheses and “for example” (or similar) when part of a sentence.

*Response: We have searched and replaced the use of ‘e.g.’ outside of parentheses as suggested.*

• In the section discussing potential limitations and drop out rates for summaries the following sentence “This may be a real effect if participants were disengaged by the technical summary.” needs tightening up (i.e., it’s not clear what “real effect” means). Suggest also linking to the need for future research examining effects of disengaging due to technical summaries.

*Response: This section has been clarified.*

General
• There are some grammatical and typographic errors in the manuscript that will need correcting prior to publication

*Response: We have run further checks for grammatical and typographical errors, which we have corrected and highlighted throughout.*
